# Epstein-Barr Virus Encephalitis Presenting As Acute Disseminated Encephalomyelitis: A Challenging Diagnostic Dilemma

**DOI:** 10.7759/cureus.48277

**Published:** 2023-11-04

**Authors:** Neelanjana Pandey, Arpan Chawala, Sameer Kandhi, Manjeet Dhallu, Sridhar Chilimuri

**Affiliations:** 1 Internal Medicine, BronxCare Health System, Bronx, USA; 2 Neurology, BronxCare Health System, Bronx, USA

**Keywords:** immunocompetent adult, acyclovir in encephalitis, intravenous immunoglobulins (ivig), epstein-barr virus, acute disseminated encephalomyelitis (adem), ebv encephalitis

## Abstract

Epstein-Barr virus (EBV) is a common human herpesvirus associated with a wide range of clinical manifestations, primarily affecting the lymphoid system. However, central nervous system (CNS) involvement, although rare, can occur and present a diagnostic challenge, particularly in immunocompetent individuals. We present a case of a 28-year-old healthy female who initially presented with a flu-like illness, her symptoms rapidly progressed, leading to neurological deficits, and altered mental status. The patient's diagnostic workup, including a viral panel and various antibodies, failed to provide a conclusive diagnosis. However, lumbar puncture revealed significant abnormalities in cerebrospinal fluid (CSF), including elevated white blood cell count and elevated CSF protein. Neuroimaging studies demonstrated non-specific findings in subcortical white matter, pontomedullary junction, and extended spinal cord lesion. Tragically, the patient's condition rapidly worsened, with diffuse cerebral edema observed on repeat imaging, leading to the patient's demise even after conventional treatment. CSF analysis, performed at an apex lab, unexpectedly returned positive for EBV PCR, indicating a diagnosis of EBV encephalitis or EBV-associated acute disseminated encephalomyelitis (ADEM).

This case highlights the challenges encountered in diagnosing EBV-associated CNS manifestations, especially in immunocompetent individuals, where these presentations are exceedingly rare. The atypical clinical course, negative initial laboratory investigations, and absence of specific radiological findings further complicated the diagnostic process. Early recognition and consideration of infectious etiologies, including EBV, in patients presenting with unexplained encephalitis or ADEM-like symptoms, are essential for timely intervention and optimal patient outcomes.

## Introduction

Encephalitis is defined by inflammation of the brain parenchyma leading to neurologic dysfunction that can be caused by infectious or non-infectious origin ​[1]​. The most common etiology is viral, which includes herpesviruses 1 and 2 (HSV-1 and HSV-2), enterovirus, and arbovirus. Other less common viruses are seasonal influenza, cytomegalovirus (CMV), Epstein-Barr virus (EBV), and human herpesvirus 6 (HHV-6)​ [2]​. EBV, also known as human herpesvirus 4 (HHV-4), is indeed a double-stranded DNA virus. It belongs to the herpesvirus family and is one of the most common viruses affecting humans. EBV primarily infects human B-lymphocytes and epithelial cells, giving rise to infectious mononucleosis, commonly referred to as “mono” or “kissing disease.” In rare instances, it can escalate into severe complications, such as EBV encephalitis, which can lead to significant neurological impairments [3]. Most of the patients present with some neurological symptoms, which can have outcomes from complete recovery to death [4]. Here, we describe a case of CNS complications due to EBV infection in a young healthy woman, who did not respond to the conventional available treatment, and eventually had a poor outcome.

## Case presentation

The patient is a 28-year-old female with a past medical history of asthma who presented to the Emergency room (ER) with complaints of retro-orbital headache, dizziness along with fever (T 100 F), chills, nausea, and multiple episodes of non-bilious vomiting for four days. She also reported sick contact with her mother at home, who had similar symptoms a few days before but denied any recent history of traveling outside New York.

Labs drawn in the ER showed leukocytosis (WBC 11.4K/µL, Normal 4-11K/µL) with the rest being unremarkable. She eventually got discharged from ED with the diagnosis of flu-like illness but returned two days later with complaints of worsening headache, dizziness, nausea, and vomiting. Vitals noted and labs drawn in the ER during this visit were within normal limits including a negative respiratory viral bio fire panel. Chest x-ray revealed no acute pathologies. However, the patient soon developed fever spikes (Tmax 102 F) while in the ED along with the presence of unsteady gait and mild bilateral dysmetria on physical examination.

CT head was obtained which was unremarkable. Lumbar puncture was carried out on day 2 with the cerebrospinal fluid (CSF) analysis showing elevated WBC 152 with 88% of neutrophils, RBC 156, and high CSF protein count 256 mg/dL along with negative VDRL, bacterial and viral cultures (Adenovirus, HSV I/II, CMV, VZV, enteroviruses). The serum HIV antibody was also negative.

**Figure 1:**
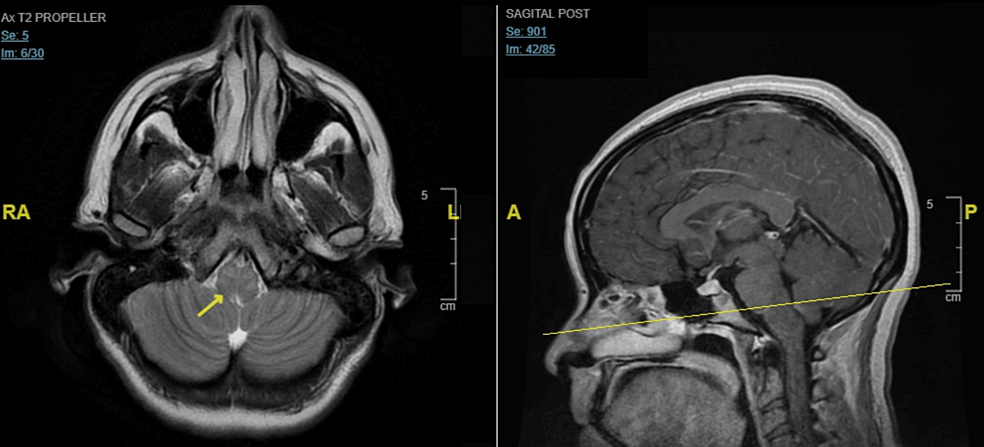
MRI brain axial T2 (right) and sagittal (left) post-contrast section

The patient was started on broad-spectrum antibiotics along with anti-viral acyclovir. The patient started to report the acute onset of diplopia, ocular ultrasound showed increased right eye optic nerve sheath diameter and papilledema. MRI head (Figure 1) showed non-specific signal abnormalities in subcortical white matter and pontomedullary junction along with mild symmetric meningeal enhancements.

Repeat lumbar puncture showed elevated opening pressure 54 cm CSF: elevated CSF WBC with lymphocytic pleocytosis 95% and a significantly up truptrendingprotein from 256 mg/dL to 404 mg/dL.

**Table 1:** CSF results on admission and during hospital course

CSF - Lumbar puncture	On admission	Hospital course	Reference Value
Colour/ Appearance	Colourless/ Clear	Colourless/ Clear	Colourless/ Clear
RBC Count	156	36	<5
WBC Count	152	85	<5
Neutrohil/ Monocyte/ Lymphocyte Count (%)	88/5/8	5/0/95	-
Glucose (mg/dl)	51	38	40-70
Protein (mg/dl)	256	404	<40
LDH (units/L)	-	167	<40
H influenza Ag/ S Pneumonie Ag/ Group B Streptococci Ag/ N Meningitidis Ag	Negative	Negative	-
Gram stain	No Organisms	No Organisms	-
Aerobic Culture	Few WBCs	No WBC, No Organism	-
Mycobacteria Culture	-	No Mycobacterium Species isolated	-
VDRL CSF	Non-reactive	Non-reactive	-
HSV DNA PCR	Not Detected	-	-
CMV DNA PCR/CMV Ab	-	Not Detected	-
West Nile Virus IgM/ IgG	-	Not Detected	-
Lyme Ab IgM/ IgG	-	No Bands	-
Cryptococcal Ag	-	Not Detected	-

On day 5 of admission, the patient had worsening mental status along with acute onset motor weakness involving upper and lower extremities, dysarthria, and worsening respiratory distress with inability to manage oral secretions requiring intubation, started on high-dose IV steroids, antibiotics, and IV immunoglobulins. An ophthalmological exam revealed bilateral papilledema for which IV acetazolamide was started. Workup for extensive serum autoimmune antibodies, serum electrophoresis, Vit B12, LFTs, IgG titers, Lyme Ab, ganglioside antibodies, acetyl-choline antibodies, HSV/mycoplasma/enterovirus/tick-borne disease antibodies were all either negative or within normal limits.

CSF analysis for MS/cryptococcal/CMV/HSV/Lyme/VZV/bacterial/fungal/mycobacterial/West Nile/Rickettsia panels was unrevealing. CT Imaging of the chest/abdomen showed no evidence of solid tumors along with a negative serum Hu Ab screen ruling out the possibility of Limbic/NMDA/other paraneoplastic-associated encephalitis. The patient was started on plasmapheresis for possible autoimmune encephalomyelitis on day 7, given the suspicion of a long lesion extending from the lower medulla to the T-spine on the MRI of the cervical spine.

**Figure 2:**
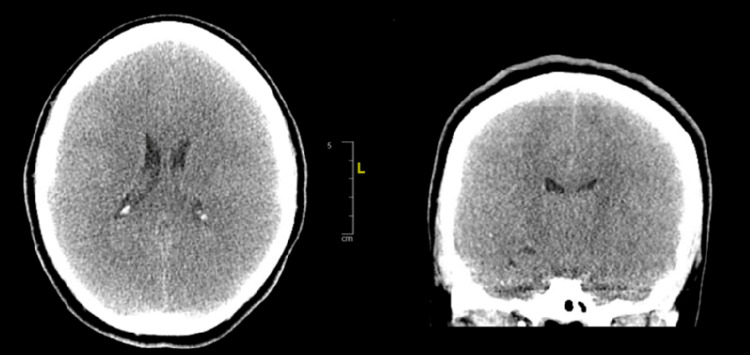
CT brain axial (right) and coronal (left) sections

While the patient was awaiting to get transferred to a tertiary neurocritical care service, became more tachycardic, tachypneic, and hypertensive on a ventilator, with new-onset seizures. Repeat CT brain (Figure 2) showed features of diffuse cerebral edema.

EEG showed multiple spikes. Aquaporin and MOG antibodies (autoimmune encephalomyelitis/NMO panel) returned as negative. The patient passed away while at the tertiary neurocritical care unit (four days following the transfer). CSF analysis sent to an apex lab returned positive for EBV PCR (EBV encephalitis vs. EBV-associated acute disseminated encephalomyelitis (ADEM) in a young immunocompetent host).

## Discussion

EBV encephalitis is most common in children and immuno-compromised adults. However, it can occur rarely in immuno-competent adults [3]. In this case report, we describe a rare case of EBV encephalitis vs. EBV-associated ADEM in young immune-competent women.

In our case, the patient presented with headache, vomiting, and vertigo sensation. The patient did not have febrile episodes before presenting but later progressed to high-grade fever and photophobia/phonophobia during the disease course, which was an unusual presentation for an EBV infection. In reported cases, EBV infection usually precedes glandular fever-like illness for two weeks before CNS involvement [4]. In most cases, EBV infection is accompanied by liver (20%) and spleen enlargement (50%), which was absent in our patient [3,5,6].

Even though EBV infection of the central nervous system (CNS) is rare (incidence rate, 0.5%-7.5% of total EBV cases, the usual course of CNS involvement is meningoencephalitis, encephalitis, cerebellitis, ADEM, transverse myelitis, Guillain-Barre syndrome, Bell’s palsy, cerebellar ataxia, and rarely daytime sleepiness [7-9]. In our case, the patient developed progressing lethargy, nuchal rigidity, and seizure episodes. Patient outcome is usually based on the involved region, but sequelae were reported maximum in thalamic (40%), and limbic system (37.5%) involvement, while the highest mortality was associated with brain-stem involvement (50%). On the contrary, normal MRI findings or cerebral hemispheric involvement were associated with a good prognosis [10].

EBV infection is usually diagnosed with serology and CSF evaluation. The presence of IgM or IgG antibodies to EBV capsids suggests acute infection but it can also be negative in early disease course [11,12]. CSF evaluation is essential in all cases of encephalitis [2]. Mononuclear count and protein content is usually elevated in CSF cytochemical analysis, while CSF PCR to detect EBV DNA is strongly useful in diagnosing EBV encephalitis in the setting of negative serology [3,13]. In our case, the CSF exam revealed elevated CSF pressure, and increased lymphocyte and monocyte count with elevated protein levels. Our patient underwent various infectious and non-infectious work-up for encephalitis, and out of that, CSF PCR for EBV DNA turned out to be positive, which put us in the dilemma of EBV encephalitis vs EBV-associated ADEM, which was also limitation to this study.

There is no standard treatment for EBV meningoencephalitis but early treatment with acyclovir and, high-dose steroids is associated with good prognoses [14]. Another study suggested the use of intravenous immunoglobulins [15]. In our case, the patient received Acyclovir, high-dose steroids, and intravenous immunoglobulins before the diagnosis of EBV encephalitis, based on presumed infectious etiology, but it did not change the disease progression and outcome [2].

## Conclusions

We encountered challenges in diagnosing EBV-associated CNS manifestations in immunocompetent individuals. The atypical clinical course, negative initial laboratory investigations, and absence of specific radiological findings can be challenging to the diagnostic process. Early recognition and consideration of infectious etiologies, including EBV, in patients presenting with unexplained encephalitis or disseminated encephalomyelitis-like symptoms, are essential for timely intervention and optimal patient outcomes.
